# Annotations

**Published:** 1888-05-19

**Authors:** 


### ANNOTATIONS.

**The Site of St. Mary's Hospital.**

LORD RANDOLPH CHURCHILL, in his speech at the St. Mary's Hospital dinner, at the Hotel Metropole on Saturday last, admitted that till that day, when Mr. Malcolm Morris took him to the hospital, he had not known where it was situated. He said that this fact showed him to be a bad Member of Parliament; but those who have sought to find St. Mary's Hospital under any but the most favourable circumstances will hesitate to endorse that statement. Lord Randolph's ignorance proves only that the hospital is situated in a most out-of-the-way and inconvenient locality, where it can be discovered only by the most persevering effort. This is certainly a blunder. There are two reasons why a hospital should be placed in a situation where it can catch the public eye; first, that the sick or hurt may know where they can find relief, and secondly, that the charitable may be constantly reminded of the existence of an institution that demands and deserves their alms. It is said that even those peripatetic topographic encyclopædias, the London cabmen and the London police, don't know where St. Mary's Hospital is situated; which means when the question is made a practical one, that when any unfortunate person meets with an accident, or falls down in a fit in the street, there is no one capable of pointing out the hospital to which he ought to be taken. The policeman makes inquiries, the cabman drives his helpless fare from street to street till he chances on the institution he seeks; but meanwhile time is being lost —time which may mean life. It is true that St. Mary's Hospital does not lack patients; on the contrary, it is forced daily to turn them from its doors from lack of room and lack of means to receive them. Still, every district should have a hospital where accidents can be received at any hour of the day and night; and that, as far as possible, all the inhabitants should know where it is. This is what the governors of St. Mary's desire, and for this purpose they intend to purchase a piece of ground facing Praed Street. They must get it; they want beds for the sick of a district whose inhabitants number a quarter of a-million. What would we say of any provincial town with that population which had only 280 beds for the sick poor among them. Yet that is all St. Mary's has space for at present. It has a medical school which is steadily increasing in size, and must be adequately provided for. It has a nursing staff, composed of refined and educated women, under Miss Medill's able charge, who are now far too poorly lodged; and there is so little room to spare that many of the servants have to sleep out of the hospital. The acquisition of the Praed Street site will do much to remove these disabilities; and it is probable that by bringing the hospital more under the public eye, it will help to bring in contributions which will more than make up for the extra expenditure involved in enlarging the building. This is the governors' hope; it is, most heartily ours also.

**Cheap Houses for Ladies.**

WHEN the history of the latter half of the nineteenth century comes to be written there will be noted, as one of its special features, the increasing importance of women in national life. We no longer hear much about the female sex—a phrase which implies a great degree of homogeneity, as if women, all and sundry, could be so labelled, and then, as far as active participation in the world and its work is concerned, be pigeon-holed and put away. The individuality of women has become a very marked thing indeed; and individuality means independence, and independence freedom. As women feel that their work is increasing in character and value they become less ready to submit to hindrances of any sort which interfere with its progress. One of the great vexations that women have long felt in London has been the want of cheap and respectable lodgings in good localities. The women who have begun to work and have not yet made their way very far up the world's ladder, the women who are studying with a view to making their living some day—having that wholesome horror of being a burden on over-weighted fathers and struggling brothers, or perhaps more remote and reluctant relations, which nobly distinguishes the gentlewomen of our time—these, and the women who have retired from life's struggle on a competence, which is, in most cases, very mode t indeed, are all desirous of finding a lodging which shall be cheap and comfortable. Till very recently it was a difficult matter. Life in a boarding-house was ghastly in its un-homelike pretence of home, and, moreover, it was expensive; but for a woman—at least a young woman—to take rooms by herself was, besides the chance of being cheated by an unscrupulous landlady, considered "not proper." Not proper! A girl of the last generation would have shrivelled under the words, and have given up on the spot all thoughts of living her individual life. A girl of ours looks the critic in the face with her honest eyes, and goes her way, proving her right to freedom by using it well. But still the lodging question was difficult, and to Lady Mary Feilding, the president of the Working Ladies' Guild, belongs the honour of solving it. She took a block of rooms originally intended for artisans, and made some slight alterations in it to fit it more or less for ladies. These rooms she let (some furnished, some unfurnished) at rents varying, according to situation, from 2*s.* 6*d.* to 4*s.* a-week. A lady could there have three rooms, forming a small flat by themselves, or she could have two, or one if her means would not afford more; but there she could live in comfort and privacy, surrounded only by women of her own stamp. A cook-housekeeper, with two or three servants under her, dwells on the ground floor. These do the roughest part of a housemaid's work, and cook a simple dinner, which the occupants may have brought to them at a very moderate price, if they so choose. Some do, but many are out all day, and either dine out or make their chief meal in the evening, in which case, unlike the old boarding-house plan, they don't pay for what they don't consume.

**An Unsavoury Subject.**

HOWEVER unsavoury the question of sewage may be in general, it is very necessary that the people of London should pay a good deal of attention to it. A city which contains a poison conduit in every street, and rivers of destructive fluid resulting from the convergence of the conduits, cannot afford to put the subject on one side merely because it is unpleasant. In human affairs nothing is more certain than the reckoning day. The landlord may sometimes be put off from Lady Day to Michaelmas, but he cannot be put off indefinitely. In like manner certain causes which are constantly at work producing certain effects, will insist upon the manifestation of their proper results sooner or later. There may be methods of checking the process, but the checks cannot be permanent obstructions. Town sewage is powerfully, terribly poisonous. That is the truth, which nobody can deny. It ought, if possible, so to be got rid of as not to contaminate either air or water. It should, in fact, be returned to the soil. Nobody in London seems to be able to devise methods of returning it to that natural and safe destination. Many persons, who perhaps do not know the difference between guano and quicklime, profess to believe that sewage is valueless as manure. If they would honestly admit that they are entirely ignorant on that point, and that the real difficulty is one of conveyance, they would be more worthy of respect. The town of Southampton has devised an inexpensive method of dealing with its sewage, and the process results in the production of a solid residuum, which farmers are glad not only to carry away, but also to pay for. Sir Henry Roscoe has the London sewage in hand for disposal, and Sir Henry Roscoe will no doubt do all that scientific chemistry can do to separate the liquid from the solid, to make the former as pure as possible before he turns it into the Thames, and to get the latter carried well out to sea. That is so far well; but the sea should not be the ultimate destination of the solid portion of the sewage. Let the purified liquid be carried out to sea by all means, or at least let it not be thrown into the river; but all the solid matter should undoubtedly be returned to the soil. The sewage question is not to be solved by chemists alone, or by engineers alone, but by men of practical capacity, who know how to make valuable things marketable, and who are able to find markets for marketable wares. London often swears by what it calls science, and entirely despises sense. It is pretty certain, however, that in this rough-and-tumble world sense without science will generally give a better account of itself than science without sense.

